# Association between subcortical nuclei volume changes and cognition in preschool-aged children with tetralogy of Fallot after corrective surgery: a cross-sectional study

**DOI:** 10.1186/s13052-024-01764-0

**Published:** 2024-09-19

**Authors:** Liang Hu, Kede Wu, Huijun Li, Meijiao Zhu, Yaqi Zhang, Mingcui Fu, Minghui Tang, Fan Lu, Xinyu Cai, Jia An, Nishant Patel, Ye Lin, Zhen Zhang, Ming Yang, Xuming Mo

**Affiliations:** 1 Department of Cardiothoracic Surgery, Children’s Hospital of Nanjing Medical University, 72 Guangzhou Road, Nanjing, 210008 China; 2 Department of Radiology, Children’s Hospital of Nanjing Medical University, 72 Guangzhou Road, Nanjing, 210008 China; 3 Medical School of Nanjing University, Nanjing, 210093 China

**Keywords:** Tetralogy of Fallot, Subcortical nuclei, Neurocognition, Preoperative cardiac structural changes

## Abstract

**Background:**

Neurocognitive disorders frequently occur in patients with cyanotic congenital heart disease (CCHD) because of the hemodynamic abnormalities induced by preoperative cardiac structural changes. We aimed to evaluate subcortical nuclei volume changes and cognition in postoperative tetralogy of Fallot (TOF) children, and analyze their relationship with preoperative cardiac structural changes.

**Methods:**

This case-control study involved thirty-six children with repaired TOF and twenty-nine healthy controls (HCs). We utilized three-dimensional (3D) T1-weighted high-resolution structural images alongside the Wechsler Preschool and Primary Scale of Intelligence-Fourth Edition (WPPSI-IV) to evaluate the cognitive differences between the TOF and HC group.

**Results:**

We observed notable differences in subcortical nuclei volume between the TOF and HC group, specifically in the left amygdala nucleus (LAM, TOF: 1292.60 ± 155.57; HC: 1436.27 ± 140.62, *p* < 0.001), left thalamus proper nucleus (LTHA, TOF: 6771.54 ± 666.03; HC: 7435.36 ± 532.84, *p* < 0.001), and right thalamus proper nucleus (RTHA, TOF: 6514.61 ± 715.23; HC: 7162.94 ± 554.60, *p* < 0.001). Furthermore, a diminished integrity of LAM ( *β*:-19.828, 95% CI: -36.462, -3.193), which showed an inverse relationship with the size of the preoperative ventricular septal defect (VSD), correlated with lower working memory indices in children with TOF.

**Conclusions:**

Our findings indicate that subcortical nuclei structural injuries possibly potentially stemming from cardiac anatomical abnormalities, are associated with impaired working memory in preschool-aged children with TOF. The LAM in particular may serve as a potential biomarker for neurocognitive deficits in TOF, offering predictive value for future neurodevelopmental outcomes, and shedding light on the neurophysiological mechanisms of these cognitive impairments.

## Introduction

Tetralogy of Fallot (TOF) represents the most prevalent form of cyanotic congenital heart disease (CCHD), comprising about 3.5% of all CHD instances [1, 2]. TOF is characterized by distinct anatomical anomalies such as ventricular septal defect (VSD), right ventricular outflow tract obstruction (RVOTO), overriding aorta (AO) and right ventricular hypertrophy. These features result in hemodynamic irregularities that precipitate a cascade of hypoxic-ischemic incidents [3]. Research indicates that TOF patients undergoing corrective surgery before reaching five years of age can achieve a 30-year survival rate of up to 90% [4]. Although surgical mortality rates have declined, survivors encounter numerous complications [5]. Notably, brain injuries during the perioperative period of TOF have garnered heightened scrutiny due to their potential link to deteriorating neurocognitive, psychosocial, and behavioral functions. Prolonged lack of intervention could detrimentally impact patients’ long-term quality of life [6–8]. According to the World Health Organization [9, 10], health-related quality of life is a multidimensional concept that integrates various facets, including physical health, psychological state, social interaction, and occupational capability. While the aspect of physical health in patients receiving comprehensive TOF repair has been profoundly studied, the domain of social cognitive function still requires further exploration.

Developmental anomalies in children with CHD have been substantially investigated [11]. Studies have suggested that children with CCHD are susceptible to neural damage during prenatal, pre-postpartum, perioperative, and postoperative phases. This damage is chiefly attributed to cerebral circulation disturbances, cardiopulmonary bypass procedures, anesthesia, reduced cardiac output, and socioeconomic factors. The prenatal and perinatal periods align with crucial stages of brain development and are deemed to present the greatest risk for neurological impairment [11–13]. The incidence of atypical cerebral alterations in children with CHD is around 10%, but this figure may escalate to 50% in those with CCHD. Magnetic resonance imaging (MRI) can reveal these aberrant cerebral modifications, which are evidenced by diminished brain size, retarded neural development, white matter injury, and cerebrovascular accidents [14–19]. Moreover, reports of delayed cortical maturation in CCHD are on the rise, primarily characterized by postponed cortical gyrification, uneven cortical depth, and diminished cortical density [20–23]. Concurrently, these children might exhibit lower intelligence quotients (IQ) and a higher incidence of behavioral issues and executive function deficiencies compared to their counterparts [24, 25]. Nonetheless, these investigations have primarily focused on preoperative and short-term postoperative morphological changes. There is a need for additional research to monitor long-term changes in subcortical nuclei and to delve deeper into the connection between these changes and cognitive functions. However, the effect of volume of subcortical nuclei changes on postoperative cognitive ability in children with TOF is still unclear. Consequently, we assessed the subcortical nuclei volume in TOF patients via MRI to appraise their cognitive capabilities, in conjunction with the anatomical alterations associated with TOF, and further scrutinized the interrelation between these factors.

## Materials and methods

### Subjects

Between November 2019 to November 2021, we enrolled 36 preschool-aged children diagnosed with TOF. Out of these, 31 patients completed the 3D T1-weighted high-resolution structural imaging examination and their data qualified for further analysis. The date without head movement or other artifacts were further analyzed. All children with TOF, who underwent surgical repair at the Children’s Hospital of Nanjing Medical University, presented no known genetic syndromes. The inclusion criteria for children with TOF included: (1) aged between 3 and 6 years; (2) absence of congenital or metabolic diseases except for TOF with pulmonary stenosis; (3) no history of mental illness or psychiatric medication; (4) no central nervous system diseases, such as tumors or trauma; (5) cardiopulmonary bypass surgeries performed before the age of 3 years; and (6) right-handedness. The control group comprised 29 healthy children (HC), matched with TOF group in terms of age, gender, and education level, without any cardiovascular and nervous system diseases. Healthy controls were recruited through the following method: (1) children who underwent regular physical examinations at the child health care clinic; (2) outpatients with transient fever who were healthy after follow-up; and (3) volunteers from the community. The inclusion criteria for healthy controls were: (1) no history of surgery; (2) no central nervous system disease; (3) no history of mental illness or psychiatric medication; (4) no congenital or metabolic disease; and (5) right-handedness. The exclusion criteria included: (1) contraindication for MRI examination and (2) refusal to participate in this study. Informed consent was obtained from the legal guardian of the children, and the protocol was approved by the Institutional Ethics Committee of Children’s Hospital, Nanjing Medical University (201907212-1).

### Multimodal MRI acquisition and processing

**Fig. 1:**
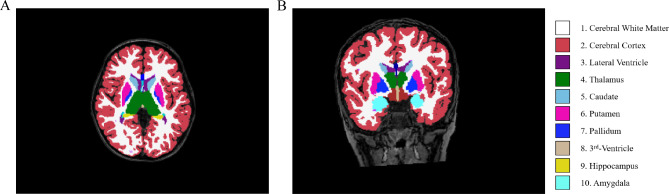
Segmentation of the subcortical structures. 3D T1-weighted image of a participant with delineation of the subcortical structures: cerebral white matter (color 1), cerebral cortex (color 2), lateral ventricle (color 3), thalamus (color 4), caudate(color 5), putamen (color 6), pallidum (color 7), 3rd -Ventricle (color 8), hippocampus (color 9) and amygdala(color 10) in the axial (**A**) and coronal (**B**) planes

All participants underwent scans during natural sleep using a 3.0 Tesla MRI system (Ingenia 3.0, Philips Healthcare, The Netherlands) in the radiology department of our hospital using a 16-channel head coil. Subjects who could not complete the MRI during natural sleep were sedated with chloral hydrate (1 ml/kg), with parental consent. Earplugs were used to reduce scanning noise, and foam was employed to minimize head motion. The scanning parameters for the 3D T1-weighted high-resolution structural images were: echo time (TE): 3.5 ms; repetition time (TR): 7.9 ms; field of view (FOV): 200 × 200 × 200 mm; slice thickness: 1 mm and acquisition time: 4 min 24 s. The scanning parameters for conventional axial T2-weighted images are as follows: TE: 110 ms; TR: 4000 ms; FOV: 200 × 200 × 119 mm; slice thickness: 5 mm and acquisition time: 1 min 28 s. Two experienced pediatric neuroradiologists, blinded to each participant’s medical history, independently reviewed all images. In cases where there was a difference of opinion, a consensus was reached through discussion. The 3D T1-weighted images were processed using FreeSurfer, V6.0 (https://surfer.nmr.mgh.harvard.edu), employing an automated recon-all pipeline that included motion correction and conform, intensity normalization, talairach transformation computation, skull stripping, removing neck, brain extraction, segment of subcortical white and gray matter, smooth, inflating, topology correction and surface deformation, spherical registration, cortical and subcortical parcellation. Subsequently, the quality of the preprocessed images was verified, including checks for skull stripping, Talairach transformation, and segmentation. Finally, the subcortical nuclei were segmented into 10 subregions (Fig. 1): left thalamus proper nucleus (LTHA), left caudate nucleus (LCAU), left putamen nucleus (LPU), left pallidum nucleus (LPA), left amygdala nucleus (LAM), right thalamus proper nucleus (RTHA), right caudate nucleus (RCAU), right putamen nucleus (RPU), right pallidum nucleus (RPA) and right amygdala nucleus (RAM).

### Assessment of neurocognition

All subjects were evaluated using the Wechsler Preschool and Primary School Intelligence Scale-Fourth Edition (WPPSI-IV). The WPPSI-IV scale testing was conducted by specifically trained personnel. During the testing process, each subject was assessed by one individual and supervised by another. Subjects were required to enter the testing room alone, with parents not permitted to accompany them. Each subject was tested in a consistent environment and given 5 min to acclimate before the formal assessment. Additionally, simple communication with the tester was encouraged to minimize potential factors that could affect test results, such as hunger, abdominal pain, or separation anxiety. The assessors were professionally trained and qualified to conduct this evaluation. Depending on the age of the subject, the assessment tasks were divided using two different scoring books: one for ages 2 Y 6 m to 3 Y 11 m and another for ages 4 Y to 6 Y 11 m.

Children aged 2 Y 6 m to 3 Y 11 m were asked to complete seven tasks. In addition to the full-scale intelligence quotient (FSIQ), the final results also included three primary indices: verbal comprehension index (VCI), visual-spatial index (VSI), and working memory index (WMI). Three subsidiary indexes: verbal acceptive index (VAI), non-verbal index (NVI), and general ability index (GAI). Meanwhile, children aged 4 Y to 6 Y 11 m completed 13 tasks. Two additional primary indices and one subsidiary index were calculated, including the fluid reasoning index (FRI), processing speed index (PSI), and cognitive efficiency index (CEI).

### Statistical analyses

Descriptive statistics were utilized to analyze the demographic characteristics of the study population. Continuous variables were expressed as means ± standard deviation or by quartile divisions. Student’s t-test was employed to compare continuous variables between two groups. Categorical variables were described using numbers and percentages. Chi-square and Fisher’s exact tests were applied to compare rates between two groups. A rank-sum test was conducted to compare ordinal data. Multivariate regression analysis was performed to examine the independent effect of subcortical nuclei changes on cognitions and preoperative cardiac structural changes. All statistical analyses were carried out using SPSS software program, version 22.0 (SPSS Inc., Chicago, IL, USA). P-value < 0.05 was considered statistically significant.

## Results

Demographic data for the TOF and HC groups is presented in Table 1. There were no significant differences in gender or family income between the two groups. A summary of essential hospitalization records for the TOF group is also provided.

**Table 1:** Characteristic information of healthy children and tetralogy of fallot children

Variables	TOF (*n* = 36)	HC (*n* = 29)	*p*-value
Age at MRI, year	3.98 ± 1.23	4.41 ± 0.91	0.104
Sex, male, %	20 (55.56%)	19 (65.52%)	0.415
Family income, ten thousand yuan per year	8.93 ± 6.78	11.97 ± 5.58	0.057
BMI, kg/m^2^	15.87 ± 1.84	17.71 ± 1.74	**< 0.001**
Age of surgery, month (*n* = 31)	12.45 ± 8.89	/	
Stay in hospital, day (*n* = 31)	25.90 ± 7.53	/	
Stay in ICU, day (*n* = 31)	5.48 ± 2.08	/	
Preoperative RVOT pressure, mmHg (*n* = 31)	66.39 ± 14.78	/	
VSD, mm (*n* = 31)	14.44 ± 3.06	/	
Overriding aorta, % (*n* = 31)	48.55 ± 7.09	/	
McGoon ratio (*n* = 31)	1.71 ± 0.33	/	
Time of surgery, min (*n* = 31)	201.77 ± 59.03	/	
Time of CPB, min (*n* = 31)	77.48 ± 18.69	/	
Time of ACC, min (*n* = 31)	54.54 ± 18.00	/	
Verbal comprehension index (*n* = 31)	87.32 ± 13.90	/	
Visual spaces index (*n* = 31)	95.32 ± 12.41	/	
Working memory index (*n* = 31)	93.13 ± 11.81	/	
Verbal acceptive index (*n* = 31)	91.13 ± 15.07	/	
Non-verbal index (*n* = 31)	92.87 ± 12.31	/	
General ability index (*n* = 31)	90.10 ± 12.35	/	
Fully scale intelligence quotient (*n* = 31)	90.35 ± 12.68	/	

Mean ± standard deviation (SD); n (percentage). Bold value represents data having statistical significance

Healthy children, HC; tetralogy of Fallot, TOF; magnetic resonance imaging, MRI; body mass index, BMI; intensive care unit, ICU; right ventricular outflow tract, RVOT; ventricular septal defect, VSD; cardiopulmonary bypass, CPB; aortic crossclamp, ACC; systolic blood pressure, SBP; diastolic blood pressure, DBP

Differences in the volume of subcortical nuclei between the TOF and HC groups are shown in Table 2. HC group exhibited higher volumes in the left caudate nucleus (LTHA) (7435.36 ± 532.84 mm^3^), left amygdala nucleus (LAM) (1436.27 ± 140.62mm^3^), right thalamus proper nucleus (RTHA) (7162.94 ± 554.60 mm^3^), right caudate nucleus (RCAU) (3777.89 ± 394.71 mm^3^), right putamen nucleus (RPU) (5215.91 ± 473.41mm^3^), right pallidum nucleus (RPA) (1794.82 ± 194.04 mm^3^), and right amygdala nucleus (RAM) (1601.27 ± 185.08 mm^3^).

**Table 2:** Volume of subcortical nuclei in patients of TOF and HC

Variables	TOF (*n* = 36)	HC (*n* = 29)	FDR
LTHA (mm^3^)	6771.54 ± 666.03	7435.36 ± 532.84	**< 0.001**
LCAU (mm^3^)	3433.84 ± 454.68	3668.40 ± 444.28	0.057
LPU (mm^3^)	4837.86 ± 676.95	5137.60 ± 551.15	0.073
LPA (mm^3^)	1725.89 ± 273.54	1876.21 ± 276.71	0.052
LAM (mm^3^)	1292.60 ± 155.57	1436.27 ± 140.62	**< 0.001**
RTHA (mm^3^)	6514.61 ± 715.23	7162.94 ± 554.60	**< 0.001**
RCAU (mm^3^)	3455.57 ± 478.70	3777.89 ± 394.71	**0.015**
RPU (mm^3^)	4846.28 ± 643.00	5215.91 ± 473.41	**0.023**
RPA (mm^3^)	1651.15 ± 230.35	1794.82 ± 194.04	**0.023**
RAM (mm^3^)	1469.20 ± 172.47	1601.27 ± 185.08	**0.015**

Mean ± standard deviation (SD). Bold value represents data having statistical significance. Data were adjusted for age and sex using FDR

Healthy children, HC; tetralogy of Fallot, TOF; left thalamus proper nucleus, LTHA; left caudate nucleus, LCAU; left putamen nucleus, LPU; left pallidum nucleus, LPA; left amygdala nucleus LAM; right thalamus proper nucleus, RTHA; right caudate nucleus, RCAU; right putamen nucleus, RPU; right pallidum nucleus, RPA; right amygdala nucleus RAM

After adjusting for sex, body mass index (BMI), age at MRI, family income, age of surgery, stay in intensive care unit (ICU), stay in hospital, time of surgery, time of cardiopulmonary bypass (CPB) and time of aortic cross-clamp (ACC), the volumes of LTHA (*β*: 0.007, 95% CI: 0.0001, 0.014), LAM CAT (*β*: 0.033, 95% CI: 0.004, 0.062), RTHA (*β*: 0.008, 95% CI: 0.002, 0.014) and RPA (*β*: 0.021, 95% CI: 0.0001, 0.041) showed associations with WMI; the volume of RTHA (*β*: 0.008, 95% CI: 0.0001, 0.016) was associated with VSI; the volume of RTH (*β*: 0.009, 95% CI: 0.002, 0.016) was linked to NVI; and the volume of RTHA (*β*: 0.008, 95% CI: 0.001, 0.016) was correlated with FSIQ (Table 3).

**Table 3:** Multiple linear regression of volume of subcortical nuclei changes and cognitions in children with TOF

Variables	VCI	VSI	WMI	VAI	NVI	GAI	FSIQ
LTHA	0.006 (-0.004, 0.015)	0.008 (-0.001, 0.016)	**0.007 (0.000**,** 0.014)**	0.003 (-0.007, 0.013)	0.007 (-0.001, 0.015)	0.007 (-0.002, 0.015)	0.008 (-0.000, 0.016)
LAM	0.013 (-0.030, 0.055)	0.004 (-0.035 0.044)	**0.033 (0.004**,** 0.062)**	0.024 (-0.018, 0.065)	0.020 (-0.016, 0.056)	0.006 (-0.033, 0.045)	0.009 (-0.029, 0.048)
RTHA	0.005 (-0.004, 0.014)	**0.008 (0.000**,** 0.016)**	**0.008 (0.002**,** 0.014)**	0.002 (-0.007, 0.011)	**0.009 (0.002**,** 0.016)**	0.007 (-0.001, 0.015)	**0.008 (0.001**,** 0.016)**
RCAU	-0.010 (-0.026, 0.007)	-0.001 (-0.017, 0.014)	-0.000 (-0.014, 0.013)	-0.009 (-0.026, 0.007)	0.003 (-0.012 0.017)	-0.002 (-0.017, 0.013)	-0.001 (-0.017, 0.014)
RPU	0.009 (-0.002, 0.019)	0.005 (-0.005, 0.016)	0.007 (-0.001, 0.015)	0.007 (-0.004, 0.018)	0.006 (-0.003, 0.016)	0.007 (-0.003, 0.017)	0.007 (-0.002, 0.017)
RPA	0.015 (-0.014, 0.043)	0.011 (-0.016, 0.037)	**0.021 (0.000**,** 0.041)**	0.007 (-0.023, 0.036)	0.016 (-0.009, 0.040)	0.019 (-0.006, 0.044)	0.023 (-0.001, 0.047)
RAM	0.004 (-0.036, 0.044)	0.018 (-0.018, 0.054)	0.020 (-0.010, 0.049)	0.011 (-0.029, 0.051)	0.023 (-0.010 0.056)	0.006 (-0.030, 0.042)	0.008 (-0.027, 0.044)

Data are shown in beta (95% CI). Bold value represents data having statistical significance. Adjusted for sex BMI, age at MRI, family income, age of surgery, stay in ICU, stay in hospital, time of surgery, time of CPB and time of ACC

Tetralogy of Fallot, TOF; confidence interval, CI; verbal comprehension index, VCI; visual-spatial index, VSI; working memory index, WMI; verbal acceptive index, VAI; non-verbal index, NVI; general ability index, GAI; full-scale intelligence quotient, FSIQ; left thalamus proper nucleus, LTHA; left amygdala nucleus LAM; right thalamus proper nucleus, RTHA; right caudate nucleus, RCAU; right putamen nucleus, RPU; right pallidum nucleus, RPA; right amygdala nucleus RAM; body mass index, BMI; magnetic resonance imaging, MRI; cardiopulmonary bypass, CPB; aortic crossclamp, ACC

Furthermore, linear regression analyses examining preoperative cardiac structural changes and subcortical nuclei volume are presented in Table 4. The size of VSD, the degree of overriding aorta and the McGoon ratio were significantly associated with LAM, RCAU and RPU and these associations were independent of sex, BMI, age at MRI and family income. Notably, the volume of LAM (*β*: -19.828, 95% CI: -36.462, -3.193) was inversely related to the size of VSD.

**Table 4:** Multiple linear regression of preoperative cardiac structural changes and volume of subcortical nuclei changes in children with TOF

Variables	Preoperative RVOT pressure, mmHg	VSD, mm	Overriding aorta, %	McGoon
LTHA	3.761 (-14.613, 22.136)	-27.101 (-111.221, 57.020)	-165.584 (-4214.907, 3883.740)	153.166 (-636.425, 942.758)
LAM	2.562 (-1.324, 6.448)	**-19.828 (-36.462**,** -3.193)**	-407.568 (879.865, 2499.070)	101.039 (-66.857, 268.936)
RTHA	2.474 (-17.560 22.509)	-3.903 (-96.202, 88.397)	503.240 (-3897.615, 4904.096)	88.791 (-772.137, 949.720)
RCAU	-3.257 (-14.464, 7.950)	2.421 (-49.509, 54.351)	**2379.803 (102.990**,** 4656.617)**	71.275 (-412.670, 555.220)
RPU	-13.859 (-29.533 1.814)	-50.245 (-124.165, 23.675)	-2074.080 (-5635.641, 1488.482)	**693.992 (36.936**,** 1351.048)**
RPA	-0.977 (-6.999, 5.045)	-20.129 (-46.637, 6.380)	-212.272 (-1534.953, 1110.408)	157.500 (-93.515 408.516)
RAM	2.027 (-2.423, 6.476)	-16.816 (-36.463, 2.830)	226.416 (-763.434, 1216.266)	161.462 (-21.271, 344.194)

Data are shown in beta (95% CI). Bold value represents data having statistical significance. Adjusted for sex, BMI, age at MRI and family income

Tetralogy of Fallot, TOF; confidence interval, CI; right ventricular outflow tract, RVOT; ventricular septal defect, VSD; left thalamus proper nucleus, LTHA; left amygdala nucleus LAM; right thalamus proper nucleus, RTHA; right caudate nucleus, RCAU; right putamen nucleus, RPU; right pallidum nucleus, RPA; right amygdala nucleus RAM; body mass index, BMI; magnetic resonance imaging, MRI

## Discussion

Our population-based follow-up study is pioneering in identifying the correlation between changes in subcortical nuclei volume and cognitive functions in preschool-aged children with TOF and the relationship between the size of VSD and the volume of subcortical nuclei change in LAM. The findings reveal a relationship among anatomical changes in TOF, subcortical nuclei volume, and cognitive behavior.

Reports have indicated delayed cortical development, compromised microstructural integrity, and abnormalities in cerebral perfusion and metabolism in the CHD population [26–28]. Functional MRI studies have also observed connectivity disorders across multiple brain regions in this demographic [29]. Research has substantiated that brain abnormalities in children with CHD correlate with negative neurodevelopmental outcomes [30]. Andropoulos et al. discovered that preoperative brain injuries (white matter damage, infarction, and hemorrhage) correlated with impaired motor and language functions in early infancy [31]. It is noteworthy that the subcortical nuclei act as gatekeepers of the central nervous system, responsible for regulating and controlling the rapid and massive information exchange between the cerebral cortex and the rest of the body, directly or indirectly affecting the change of cognitive behavior. Consequently, drawing on our results and existing literature, we hypothesize about the mechanisms underlying reduced WMI due to diminished subcortical nuclei volume in postoperative pre-school-aged children with TOF. Our study demonstrates a close correlation between the volume of LAM and the reduction in WMI, which suggests that the subcortical nuclei of TOF patients are related to postoperative cognitive and behavioral changes. Hence, it is crucial to prioritize the monitoring and protection of these subcortical nuclei.

Additionally, studies have linked reduced brain volume in infants and adolescents with CHD to deficits in cognitive, motor, and language development [32]. Furthermore, diffusion tensor imaging has revealed that delayed maturation of white matter microstructures in adolescents with CHD can adversely affect working memory, attention, and learning capabilities [32]. Children with TOF experience reduced oxygen delivery and consumption [32], making their cerebral cortex especially vulnerable to related adverse effects [20]. The primary microstructural changes in the hypoxic brain include impaired dendritic branching of neurons [33] and suppressed formation of glial cells [34–36].

The amygdala, a key structure within the limbic system, is crucial for emotional and cognitive functions [37]. Amygdala dysfunction is associated with a range of neurodevelopmental disorders and psychological conditions [38–40], including depression [41], social anxiety [42], post-traumatic stress disorder [43], dementia [44], and schizophrenia [45]. Earlier research has also shown the amygdala’s role in higher cognitive functions [39, 46], including memory [47–50], learning [51, 52], decision-making [53], reward regulation [54], and intelligence [46, 55]. Recently, more studies have identified connections between the amygdala and various memory functions, such as emotional memory processing, memory consolidation, working memory, state-dependent memory retrieval, autobiographical memory encoding/recall, and episodic memory formation/retrieval [56–60]. Although often considered a single entity, it’s important to recognize that the amygdala comprises multiple nuclei, each with unique roles and connections within the limbic system and the broader brain network.

A negative correlation was found between the size of the VSD and changes in LAM volume, while positive correlations were observed between the aorta and RCAU volume changes, and between the McGoon index and RPU volume changes. These anatomical variations correlate with the extent of ischemia and hypoxia experienced by children with TOF. This aligns with existing literature suggesting that hemodynamic irregularities in TOF are primary contributors to brain structural alterations, particularly damage to subcortical nuclei. Thus, preoperative cardiac structural factors in TOF patients play a role in forecasting postoperative cognitive outcomes. Even after correction of TOF’s intracardiac malformations, careful attention must be given to the potential for brain injury post-surgery. Meanwhile, postoperative working memory training for children with TOF should be intensified. In our study, preoperative cardiac structural changes appear to predict adverse neurodevelopmental outcomes. A large sample size may provide a more definitive determination of the link between cortical structure and neurodevelopmental outcomes in children with TOF.

This study has the following highlights: 1) The long-term cognitive function of the children with TOF was analyzed and studied. 2)MRI data is relatively stable, and we are strict on MRI data screening, which greatly ensures the integrity and reliability of data. 30) We carried out a single blind test on the experimenters who conducted the scale evaluation, and a professional supervised the implementation process to ensure the authenticity of the scale data. Nevertheless, this study has several limitations. (1) The sample size included in the analysis was limited. Being an exploratory study, we endeavored to include as many eligible cases as feasible. We aim to incorporate additional cases for validation in the future to enhance the reliability and generalizability of our findings. (2) The study lacked cognition assessments for healthy controls, as parents reported normal intelligence levels and declined cognitive testing for their children. This presented an unavoidable challenge within the scope of our research. Consequently, our study concentrated on examining brain MRI discrepancies between healthy controls and TOF subjects, exploring the potential correlations with cognitive function, and investigating underlying causes. We intend to conduct standardized tests on healthy participants in future studies. (3) An adult template was used for image normalization, which could introduce bias and limitations, potentially overlooking significant inter-population differences. In the future, we will integrate perioperative risk factors and neuroimaging analyses to identify a more suitable age-specific template. (4) While this study represents a single follow-up, conducting multimodal brain MRI and neurocognitive testing at various time points pre- and post-surgery is crucial for a deeper understanding of morphological changes and the cognitive impairment process in TOF patients.

## Conclusion

This study concludes that preschool children with TOF face a heightened risk of cognitive impairment, corroborating evidence from existing research. The study found a strong association between reduced WMI in preschoolers post-TOF surgery and morphological damage to the left amygdala, indicating that pre-correction brain damage in TOF children persists in influencing cortical development and cognitive abilities during the preschool years. Our findings propose that changes in the volume of subcortical nuclei in LAM could serve as a potential biomarker for neurocognitive deficits in TOF and may predict future neurodevelopmental outcomes.

## Data Availability

Please contact the author for data requests.
